# States of Aggregation and Phase Transformation Behavior of Metallosurfactant Complexes by Hexacyanoferrate(II): Thermodynamic and Kinetic Investigation of ETR in Ionic Liquids and Liposome Vesicles

**DOI:** 10.3390/biomimetics7040221

**Published:** 2022-11-30

**Authors:** Karuppiah Nagaraj, Subramanian Sakthinathan, Te-Wei Chiu, Subramaniam Kamalesu, Snehal Lokhandwala, Nikhil M. Parekh, Chelladurai Karuppiah

**Affiliations:** 1 SRICT, Institute of Science and Research, UPL University of Sustainable Technology, Block No. 402, Valia Rd., Vataria, Ankleshwar 393135, Gujarat, India; 2 Department of Materials and Mineral Resources Engineering, National Taipei University of Technology, No. 1, Section 3, Chung-Hsiao East Road, Taipei 106, Taiwan; 3 Department of Chemistry, University Institute of Science, Chandigarh University, Gharuan, Mohali 140413, Punjab, India; 4 SRICT, Department of Environmental Science & Technology, UPL University of Sustainable and Technology, Block No. 402, Valia Rd., Vataria, Ankleshwar 393135, Gujarat, India; 5 Battery Research Center of Green Energy, Ming Chi University of Technology, New Taipei City 24301, China

**Keywords:** liposome, ionic liquids, phase transition, metallosurfactants, hydrophobic effect, outer-sphere electron transfer

## Abstract

Electronic absorption spectroscopy was used to study the ETR of surfactant–cobalt(III) complexes containing imidazo[4,5-f][1,10]phenanthroline, dipyrido[3,2-d:2′-3′-f]quinoxaline and dipyrido[3,2-a:2′,4′-c](6,7,8,9-tetrahydro)phenazine ligands by using ferrocyanide ions in unilamellar vesicles of dipalmitoylphosphotidylcholine (DPPC) and 1-butyl-3-methylimidazolium bromide ((BMIM)Br), at different temperatures under pseudo-first-order conditions using an excess of the reductant. The reactions were found to be second-order and the electron transfer is postulated as occurring in the outer sphere. The rate constant for the electron transfer reactions was found to increase with increasing concentrations of ionic liquids. Besides these, the effects of surfactant complex ions on liposome vesicles in these same reactions have also been studied on the basis of hydrophobicity. We observed that, below the phase transition temperature, there is an increasing amount of surfactant–cobalt(III) complexes expelled from the interior of the vesicle membrane through hydrophobic effects, while above the phase transition temperature, the surfactant–cobalt(III) complexes are expelled from the interior to the exterior surface of the vesicle. Kinetic data and activation parameters are interpreted in respect of an outer-sphere electron transfer mechanism. By assuming the existence of an outer-sphere mechanism, the results have been clarified based on the presence of hydrophobicity, and the size of the ligand increases from an ip to dpqc ligand and the reactants become oppositely charged. In all these media, the ΔS^#^ values are recognized as negative in their direction in all the concentrations of complexes employed, indicative of a more ordered structure of the transition state. This is compatible with a model in which these complexes and [Fe(CN)_6_]^4−^ ions bind to the DPPC in the transition state. Thus, the results have been interpreted based on the self-aggregation, hydrophobicity, charge densities of the co-ligand and the reactants with opposite charges.

## 1. Introduction

During the last few decades, rapid advances in our understanding of surface phenomena have taken place. However, the significance of surface science has been recognized for more than a century. Surfactants are used as ligands [1] that dramatically decrease the interfacial free energy of solids and liquids [2]. Surfactants are amphipathic molecules, i.e., they possess hydrophilic and hydrophobic regions [3], with a long hydrocarbon tail and moderately small ionic or polar head group. Surface-active agents play major roles in chemistry, biology, and pharmaceuticals [4]. It has been observed that both the oxidation and reduction of micellar medium can be influenced by hydrophobic and electrostatic forces and, for a given set of reactions, the observed rate depends on the extent of the association between the micellar aggregation and the reactants [5]. In recent times, there have been reports on surfactant metal complexes of various natures and their micellar aggregation properties [6].

The electron transfer based on the outer sphere among first-row metal complexes plays a vital role both in vivo [5] and in the treatment of molecular-scale devices, such as logic gates and molecular wires [7]. The alterations of the electron transfer reactions on the outer-sphere environment complex are attributable to the variation of counter ion concentrations [8]. Gaswick et al. have reported that the [Fe(CN)_6_]^4−^ can reduce some pentamminecobalt(III) complexes to cobalt(II) via an outer-sphere electron transfer step [9] and they have also reported that the substituted pentamminecobalt(III) complexes could be reduced by [Fe(CN)_6_]^4−^ with the development of an ion pair [10]. There are many reports available on electron transfer between metal complexes and ferrocyanide ions [11,12,13,14]. A. R. Mustafina et al. [15] have studied the electron transfer reaction of outer-sphere combination of p-sulfanotothiacalix[4]arene and some cobalt-based metal complexes. The ion pairing of the complexes with macrocycle STCA accelerates the [Fe(CN)_6_]^4−^ cobalt(III) electron transfer reactions. A. J. Miralles et al. [16] have explored the outer-sphere reductions of cobalt and ruthenium-based pyridinepentammine complexes by [Fe(CN)_6_]^4−^. A. A. Holder has reported the mechanistic investigation of the reduction of the molybdatopentamminecobalt(III) ion by aqueous [Fe(CN)_6_]^4−^ and aqueous sulfite [17]. A. P. Szecsy and A. Haim [18] have studied the mechanism of the reaction which takes place in the imidazolate bridges intramolecular electron transfer between pentacyanoferrate(II) and pentammine cobalt(III) complexes containing imidazole and its conjugate base. Jing-Jer Jwo et al. [19] have performed intramolecular electron transfer between the same reactant and pentammine cobalt(III) mediated by various 4,4′-bipyridines. M. Martinez et al. [20] have reported the outer-sphere reactions of (N)_5_-macrocyclic cobalt(III) complexes. Several studies have been conducted to resolve the dependence of electron transfer on different environments, such as DNA vesicles and micelles [21,22,23,24,25], and have received plenty of interest over the last several decades. An amount of research has been devoted to the interaction of amphiphilic molecules with phospholipid vesicles membrane models due to the partition equilibrium between the bilayers and the aqueous phase; this integration involves complex perturbations in the physical properties of vesicle membranes, which pertain to the type and amount of surfactant [26]. It is evident that surfactant complexes are marked by their dual nature consisting of the hydrocarbon component (hydrophobic) and the ionic component (hydrophilic), which is responsible for the self-aggregation process in solution [27].

Vesicles are used for gene therapy in preclinical and clinical trials and have also proven to be beneficial carriers for the delivery of genes to cultured cells [28]. Surfactant–vesicle systems are good models for cell membrane solubilization studies [29]. In the past few years, ionic liquids (ILs) have generated substantial interest as potential new media for nanostructure construction materials, electrochemistry, organic synthesis and catalyst supports [30,31]. From a fundamental prospect, ionic liquids facilitate solvatophobic interactions, which are present among ionic liquids and surfactants, enhancing the solvation characteristics of the ionic liquid–surfactant system [32,33,34]. The outer-sphere electron transfer reactions of innumerable metallomicelles have been developed in our laboratory [35,36,37,38]. We have reported that both the oxidant and reductant are cations on the electron transfer between surfactant complexes with iron(II) in DPPC [35,36,37,38,39]. In the present work, we present our results on the influence of phase transition behavior in ionic liquids and liposome vesicles on the outer-sphere electron transfer between surfactant complexes containing aromatic diimine ligands and ferrocyanide ions.

## 2. Experimental Section

### 2.1. Materials

The Disodium salt of EDTA, sodium nitrate and potassium ferrocyanide were acquired from Fluka, Germany, and were used without further purification. All the solvents used were of analytical grade only. The liposome vesicles and ionic liquids were acquired from Sigma–Aldrich, India, and were used as such. Phosphate buffer solutions were prepared with the help of sodium phosphate and sodium dihydrogen orthophosphate. All solvents used were of analytical grade. To prepare buffer solutions, sodium phosphate and sodium dihydrogen orthophosphate were used.

### 2.2. Methods

#### 2.2.1. Preparation of Reductant/Oxidant

**Scheme 1:**
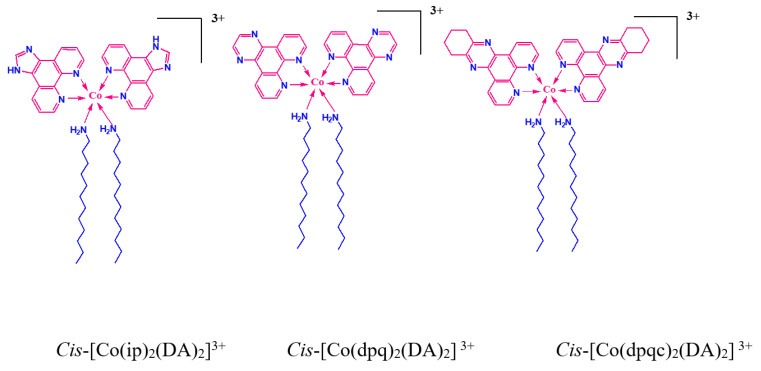
The structure of surfactant–cobalt(III) complexes.

The surfactant–cobalt(III) complexes, cis-[Co(ip)_2_(C_12_H_25_NH_2_)_2_]^3+^, cis-[Co(dpq)_2_(C_12_H_25_NH_2_)_2_]^3+^ and cis-[Co(dpqc)_2_(C_12_H_25_NH_2_)_2_]^3+^ used as oxidants were synthesized as reported in our earlier work [40]. The structure of complexes is shown in Scheme 1.

#### 2.2.2. Nature of the Reaction

On mixing [Fe(CN)_6_]^4−^ and our surfactant–cobalt(III) complexes in the aqueous solution, a precipitate was formed and, therefore, homogeneous kinetic measurements were precluded. However, when Na_2_H_2_EDTA was present in the solution, no precipitate was formed during the reaction and therefore all the experiments were carried out in the presence of Na_2_H_2_EDTA. A disodium salt of ethylenediamine tetra acetic acid acted as a sequestering agent to eradicate cobalt(II) and prevented the precipitation of the cobalt(II) ion as a hexacyanoferrate salt.

#### 2.2.3. Liposome Preparation

In the present study, unilamellar vesicles (ULV) were used and these were prepared by ethanol injection [41]. A solution of the lipid in ethanol was injected promptly into the buffer with the help of a fine needle and sustained at 50 °C under optimized conditions. The volume of ethanol injected was always <1% *v/v* in order to avoid any damage to the liposome. This method gave rise to a small unilamellar vesicle (SUV) in the size range of ~15–25 nm [42].

#### 2.2.4. Kinetic Measurements

The rate of the reaction was measured spectrophotometrically using a Shimadzu-1800 UV–Vis. spectrophotometer equipped with a water Peltier system (PCB 150). The temperature was controlled within ±0.01 °C. A solution containing the desired concentration of potassium ferrocyanide, sodium nitrate, disodium ethylenediamine tetraacetate (Na_2_H_2_EDTA), liposome/ionic liquids in oxygen-free water was placed in a 1 cm cell, which was then covered with a serum cap fitted with a syringe needle. This cell was placed in a thermostated compartment in the spectrophotometer and then the solution containing surfactant–cobalt(III) complexes was added anaerobically using the syringe, and then all increases in the absorbance of the oxidant observed at 470 nm were recorded as a function of time. The ionic strength was maintained at 1.0 mol dm^−3^ in all runs using NaNO_3_. The second-order rate constant, k, for the [Fe(CN)_6_]^4−^ reduction of the cobalt(III) complexes defined by -d[Co(III)]/dt = k[Co(III)][Fe(CN)_6_]^4−^ was calculated from the concentration of [Fe(CN)_6_]^4−^ and the slope of the pseudo-first-order plot of log(A_t_-A_∞_) versus time plot, which is equal to -k[Fe(CN)_6_]^4−^/2.303, where A_t_ is the absorbance at time t, A_∞_ is the absorbance after all the cobalt(III) complexes have been reduced to cobalt(II), and k is the second-order rate constant. Generally, the value of A_∞_ was measured at times corresponding to ten half-lives. All the first-order plots were significantly linear for at least five half-lives, with a correlation coefficient of >0.999. Each rate constant declared is the average result of triplicate runs. Rate constants attained from consecutive half-life values within a single run agreed to within ±5%.

## 3. Results and Discussion

### 3.1. Kinetics of Outer-Sphere Electron Transfer Reaction

**Scheme 2:**
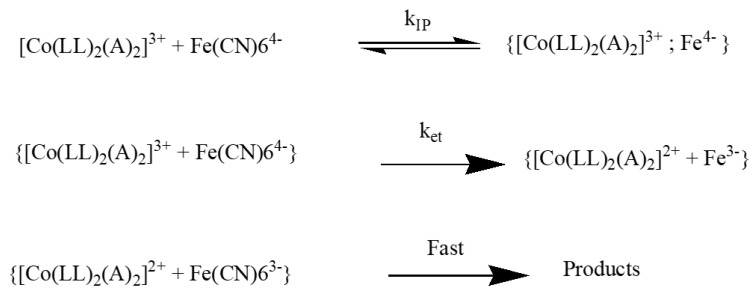
Electron transfer reaction of surfactant complexes by ferrocyanide ions. LL = ip, dpq and dpqc; A = Dodecylamine.

The reduction of the surfactant–cobalt(III) complexes, cis-[Co(ip)_2_(C_12_H_25_NH_2_)_2_]^3+^, cis-[Co(dpq)_2_(C_12_H_25_NH_2_)_2_]^3+^ and cis-[Co(dpqc)_2_(C_12_H_25_NH_2_)_2_]^3+^ using ferrocyanide ions proceeds to produce aqueous cobalt. This reaction is to be outer-sphere, by comparing the pathway of similar electron transfer reactions reported in the literature [43,44,45,46]. In the present study, these cobalt(III) complexes cannot be substituted, due to the non-availability of a coordination site for an inner-sphere precursor complex. The most favorable mechanism for the second-order reaction is an outer-sphere electron transfer process which consists of three elementary steps: ion pair formation (k_ip_), electron transfer (k_et_), and product dissociation in Scheme 2.

### 3.2. Effect of Electron Transfer Reaction in Vesicles and Ionic Liquid Media

**Figure 1:**
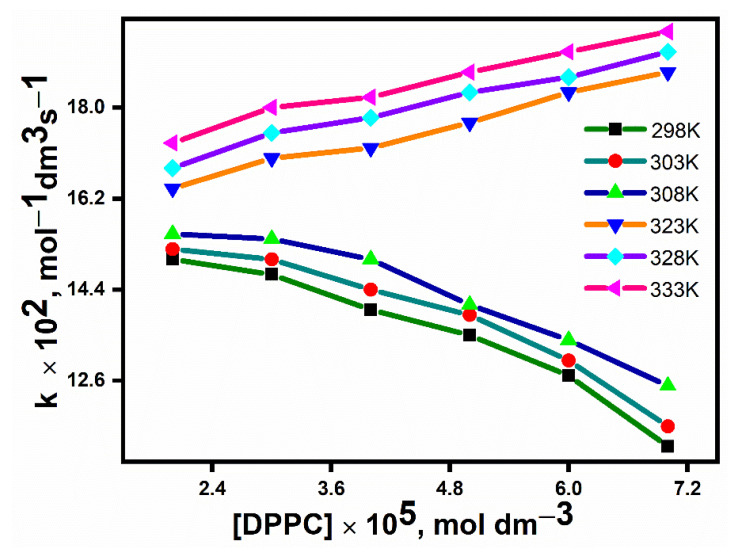
Plot of k against DPPC for cis-[Co(ip)_2_(C_12_H_25_NH_2_)_2_](ClO_4_)_3_ under various temperatures; cis-[Co(ip)_2_(C_12_H_25_NH_2_)_2_](ClO_4_)_3_ = 4 × 10^−4^ mol dm^−3^, μ = 1.0 mol dm^−3^, [Fe(CN)_6_]^4−^ = 0.01 mol dm^−3^.

**Scheme 3:**
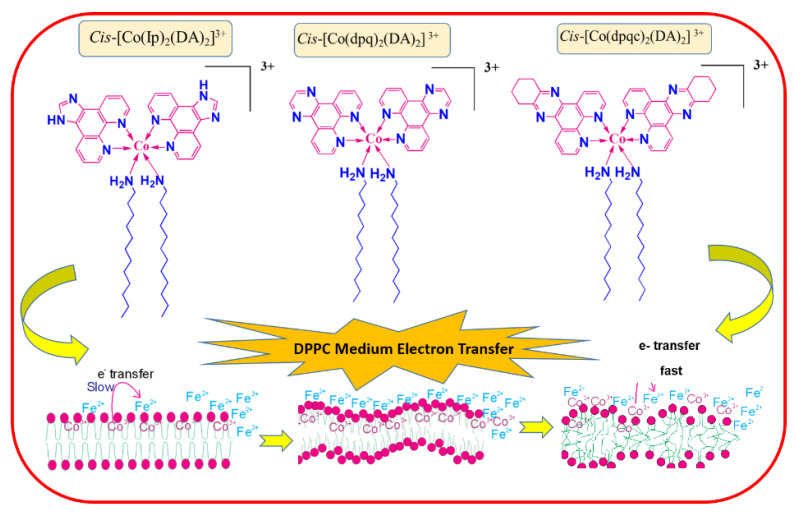
DDPC-mediated ETR of surfactant–cobalt(III) complexes.

The electron transfer reaction of surfactant–cobalt(III) complexes, cis-[Co(ip)_2_(C_12_H_25_NH_2_)_2_]^3+^, cis-[Co(dpq)_2_(C_12_H_25_NH_2_)_2_]^3+^ and cis-[Co(dpqc)_2_(C_12_H_25_NH_2_)_2_]^3+^ and ferrocyanide ions in the presence of DPPC and ionic liquid mediums have been examined at different temperatures. The rate constants are provided in Table 1 and Supplementary Information (SI) Tables S1 and S2, and the plots of k against various concentrations of DPPC are shown in Figure 1 and SI Figures S1 and S2. Comparing previous studies of electron transfer in the same liposome vesicles media, results are rather different where some of the reactants had the same charges. In the present system, comprising those of opposite charges, we have ascertained two propensities in the characteristics of the reaction with an increase in the concentration of liposome vesicles as shown in Scheme 3.

#### 3.2.1. Effect of DPPC

Because of its amphiphilic nature, DPPC undergoes spontaneous aggregation in aqueous solutions. This leads to the formation of a three-dimensional closed bilayer structure known as a vesicle [47]. When the temperature is increased, these vesicles of DPPC undergo phase transitions at 40 °C [48], from the gel phase to the liquid crystalline phase. The effect of DPPC vesicles (2.0 × 10^−5^ to 7.0 × 10^−5^ mol dm^−3^) on the kinetics of outer-sphere electron transfer between the surfactant–cobalt(III) complexes cis-[Co(ip)_2_(C_12_H_25_NH_2_)_2_]^3+^, cis-[Co(dpq)_2_(C_12_H_25_NH_2_)_2_]^3+^ and cis-[Co(dpqc)_2_(C_12_H_25_NH_2_)_2_]^3+^ and [Fe(CN)_6_]^4−^ has been investigated at various temperatures. As we have used the ethanol injection method, the reaction medium of these electron transfer reactions should contain only unilamellar vesicles. We observed two trends in the behavior of the rate constants with varying concentrations of DPPC. Below the phase transition temperature, the rate constants decrease with increasing DPPC, whereas above this temperature the rate constants increase with DPPC. These trends were observed for both the surfactant–cobalt(III) complexes used in the present study. It is well known that when a surfactant is added to an aqueous medium containing lipid membranes, the interaction between surfactant and lipids takes place in three ways: part of the added surfactant is inserted into the outer membrane leaflet; the surfactant molecules equilibrate between the outer and inner leaflets of the vesicle; and the inner leaflet equilibrates with the interior of the vesicle [49,50]. Below the phase transition temperature, the lipid is very rigid (Scheme 3), so our surfactant–metal complexes are tightly bound to the membrane DPPC, mostly at the inner membrane leaflet. As the concentration of DPPC is increased, more of the surfactant–cobalt(III) complex will be accumulated into the DPPC interior, whereas [Fe(CN)_6_]^4−^ will be at the outer surface, so the rate constant decreases (Table 1 and SI Tables S1 and S2). However, beyond the phase transition temperature, the rigidity of the DPPC membrane is low; so when the concentration of DPPC is increased, more of the surfactant–cobalt(III) complex molecules will move from the membrane interior to the outer surface where the concentration of [Fe(CN)_6_]^4−^ is also high, causing the rate constant to increase. Additionally, the phase transition may favorably affect the reorganization energies and the free energy barrier associated with electron transfer [51]. As a result, the higher hydrophobicity of complex *Cis*-[Co(ip)_2_(C_12_H_25_NH_2_)_2_]^3+^ gives a higher second-order rate constant compared to *Cis*-[Co(dpq)_2_(C_12_H_25_NH_2_)_2_]^3+^ which is, in turn, higher than that of *Cis*-[Co(dpqc)_2_(C_12_H_25_NH_2_)_2_]^3+^.

#### 3.2.2. Effect of Ionic Liquids

**Figure 2:**
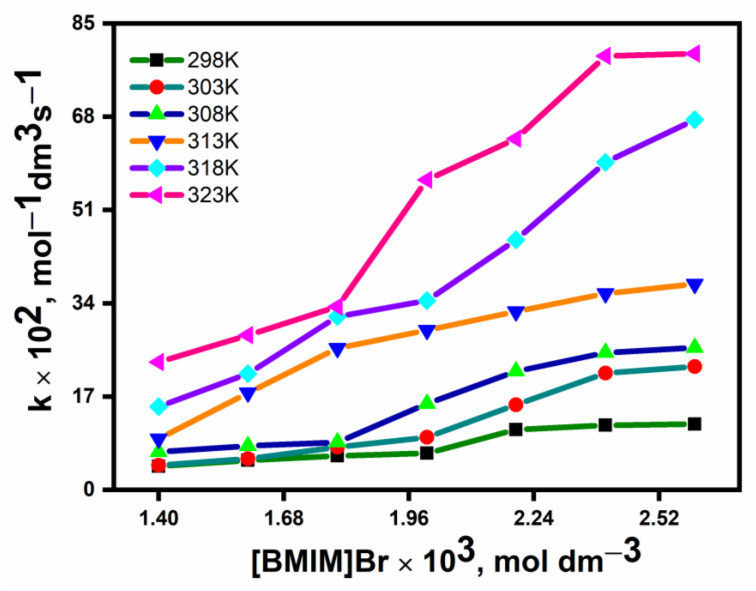
Plot of k against [BMIM]Br for cis-[Co(ip)_2_(C_12_H_25_NH_2_)_2_](ClO_4_)_3_ at various temperatures; cis-[Co(ip)_2_(C_12_H_25_NH_2_)_2_](ClO_4_)_3_ = 4 × 10^−4^ mol dm^−3^, μ = 1.0 mol dm^−3^, [Fe(CN)_6_]^4−^ = 0.01 mol dm^−3^.

The electron transfer reaction of the same complexes in the presence of ionic liquids has been studied. The effect of ionic liquid mediums on the kinetics of electron transfer regarding the oxidants and ferrocyanide ions has been examined at different temperatures. The observed second-order rate constants are given in Table 2 and SI Tables S3 and S4 and the plot of k against various concentrations of ionic liquids is shown in Figure 2 and SI Figures S3 and S4 for the above reaction, at various temperatures. As seen from these tables, the rate constant of the reaction continues to increase with an increase in the concentration of ionic liquids from 1.4 × 10^−3^ to 2.6 × 10^−3^ mol dm^−3^. As the cation of the ionic liquid used has an inherent amphiphilicity, it can interact with the long aliphatic double chain of the surfactant–cobalt(III) complexes, meaning that the specific structures before and after aggregation consist of well-aligned cation–anion aggregates. In the initial aggregation, the surfactant complexes and [Fe(CN)_6_]^4−^ ions were far apart from the ionic liquid pool, so the rate constant did not increase significantly. After aggregation, the surfactant complexes and [Fe(CN)_6_]^4−^ ions moved toward the ‘‘ionic liquids pool’’ surface. As a result of charge neutralization or charge creation, a marginal contribution to the activation volume is expected. Thus, the ionic liquids consisting of charged ions should be energetically favored, which could lead to an increase in the reaction rate with increasing concentrations of ionic liquid reactants existing in small volumes, leading to a higher rate and lower activation energy. This aggregation leads to higher local concentration of reactants leading to an increase in the rate of the reaction. Hence, the rate of the outer-sphere electron transfer reaction of the present study increases with an increase in the concentration of the ionic liquid. As changes in amphiphilicity of the transition state can cause large effects in terms of electrostriction/solvation in ionic liquids [52], the ionic liquid mediums facilitates more aggregation of the surfactant–cobalt(III) complexes, which increases with an increase in the concentration of the ionic liquid. As seen from Table 2 and SI Tables S3 and S4, the rate constant values of the surfactant–cobalt(III) complexes (Figure 2 and SI Figures S3 and S4) of the present study are very much different from one another. This difference in the rate constant values between the surfactant–cobalt(III) complexes of the present study are explained as follows: the rate constant value of the surfactant–cobalt(III) complexes containing ip and dpq ligands is lower than that of the corresponding surfactant–cobalt(III) complexes containing dpqc ligands in all the initial concentrations studied. Due to high hydrophobicity of dpqc-ligands-containing complex, the numbers of micelles formed from these complex molecules are more than those of ip- and dpq-containing complexes at the same concentration values. Hence, dpqc-containing complexes enhance the overall rate of the reaction. Among these surfactant–cobalt(III) complexes containing ip, dpq and dpqc ligands, the rate constant values of the dpqc complexes are the highest due to the greater hydrophobicity of the ligand. In the previous reports [36] of the electron transfer reaction in this media with Fe^2+^, it was established that amphiphilicity influenced the reaction rates. On comparing the previous reports of rate constant to those of the present study, the reaction rate is increased due to the increase in size of the amine ligand which increases the amphiphilicity of the oxidants which speeds up aggregation, leading to higher reaction rates of the outer sphere.

### 3.3. Oxidants with Self-Aggregation-Forming Capacity

The self-aggregation of each complex indicates its respective self-aggregation forming capacity. Lower critical micelle concentration value indicates higher aggregation-forming capacity. The difference in the self-aggregation between various surfactant–cobalt(III) complexes in the present study (Table 2 and SI Tables S3 and S4) are explained as follows: for all types of complexes, it has been observed that the critical micelle concentration value of each double-chain oxidant is lower than the respective single-chain surfactant complexes. The CMC value of each of the surfactant–cobalt(III) complex is lower than the respective single-chain surfactant complexes. Because of these extending the aromaticity of ligands, the oxidants become more aggregated, which increases the possibility of these oxidants forming self-aggregation. The order of aggregation-forming capacity of the oxidants is as follows: *Cis*-[Co(ip)_2_(C_12_H_25_NH_2_)_2_]^3+^ < *Cis*-[Co(dpq)_2_(C_12_H_25_NH_2_)_2_]^3+^ < *Cis*-[Co(dpqc)_2_(C_12_H_25_NH_2_)_2_]^3+^.

### 3.4. Activation Parameters

The effect of temperature on the rate was studied at 298, 303, 308, 313, 318, and 323 K for all rate constant tables for DPPC and ionic liquid mediums in order to obtain the activation parameters for the reaction of oxidants and the reductant of ferrocyanide ions. Figure 2 and SI Figures S3 and S4 show that increasing the temperature rate constant increases in ionic liquids, and Figure 1 and SI Figures S1 and S2 show that increasing the temperature (above phase transition) increases the reaction rate, while it decreases the reaction rate of electron transfer reactions in DPPC at below-phase-transition temperatures.

### 3.5. Isokinetic Plots

**Figure 3:**
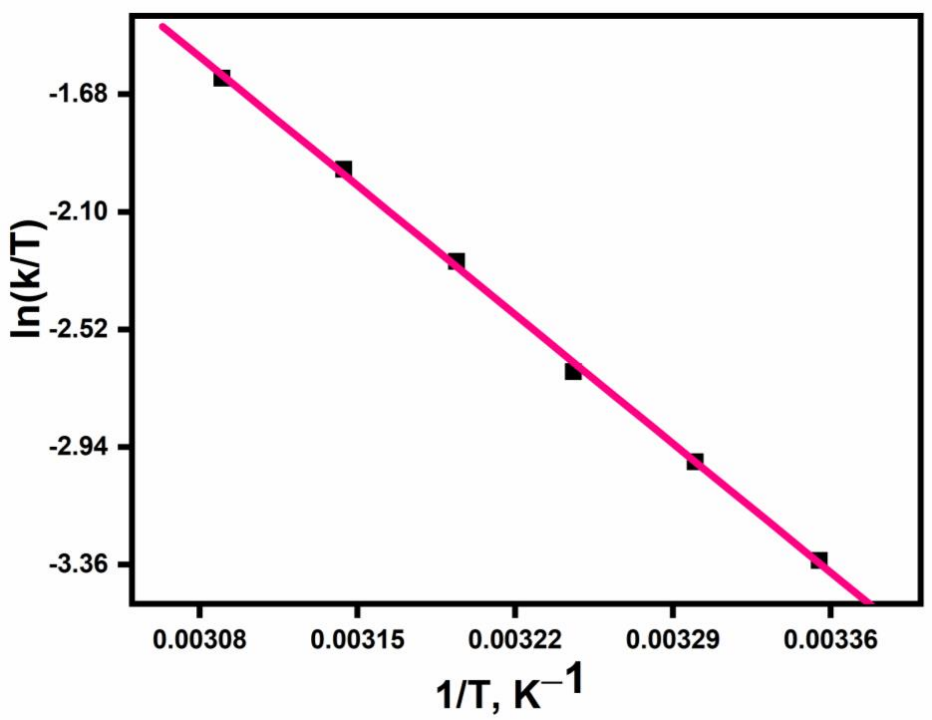
Eyring plot for cis-[Co(ip)_2_(C_12_H_25_NH_2_)_2_](ClO_4_)_3_ in DPPC medium. [Complex] = 4 × 10^−4^ mol dm^−3^; [Fe^2+^] = 0.01 mol dm^−3^; [μ] = 1.0 mol dm^−3^.

**Figure 4:**
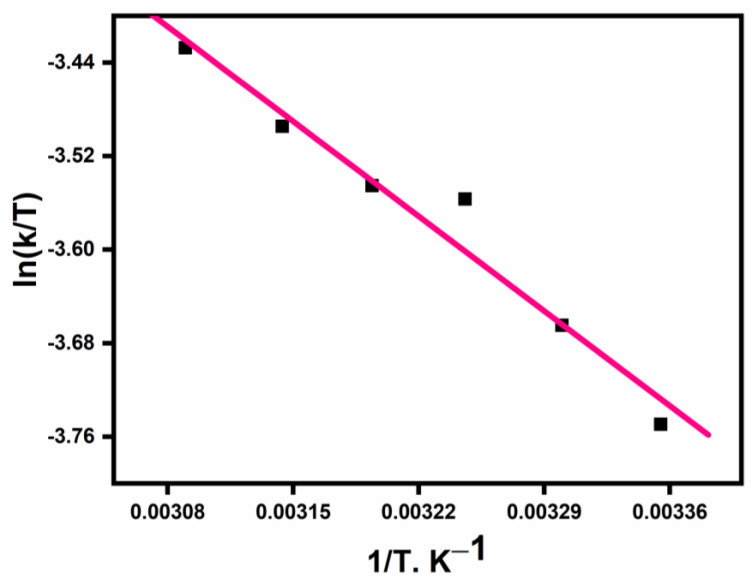
Eyring plot for cis-[Co(ip)_2_(C_12_H_25_NH_2_)_2_](ClO_4_)_3_ in [BMIM]Br medium. [Complex] = 4 × 10^−^^4^ mol dm^−^^3^; [Fe(CN)_6_]^4^^−^ = 0.01 mol dm^−^^3^; [μ] = 1.0 mol dm^−^^3^.

**Figure 5:**
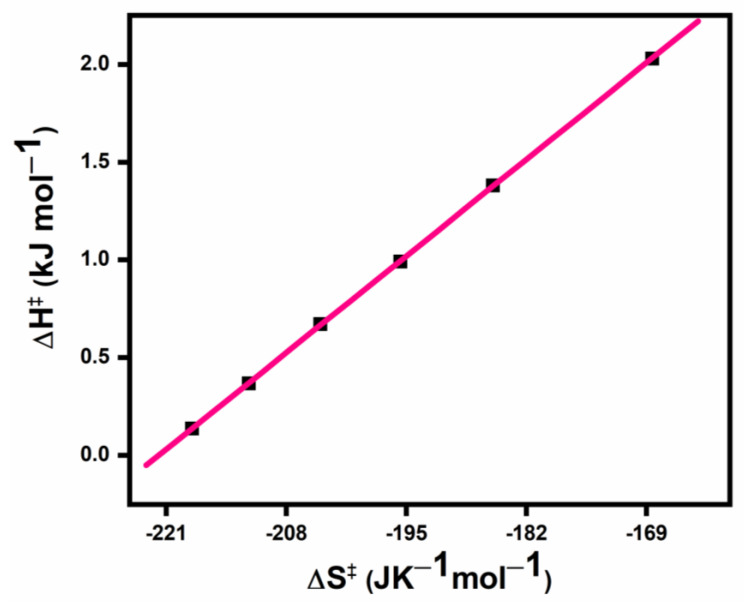
Isokinetic plot of the activation parameters for the reduction of cis-[Co(ip)_2_(C_12_H_25_NH_2_)_2_](ClO_4_)_3_ by ion(II) in DPPC medium. [Complex] = 4 × 10^−4^ mol dm^−3^; [Fe^2+^] = 0.01 mol dm^−3^; [μ] = 1.0 mol dm^−3^. (Entropy and Enthalpy of activation is symbolized ΔS^‡^ and ΔH^‡^).

**Figure 6:**
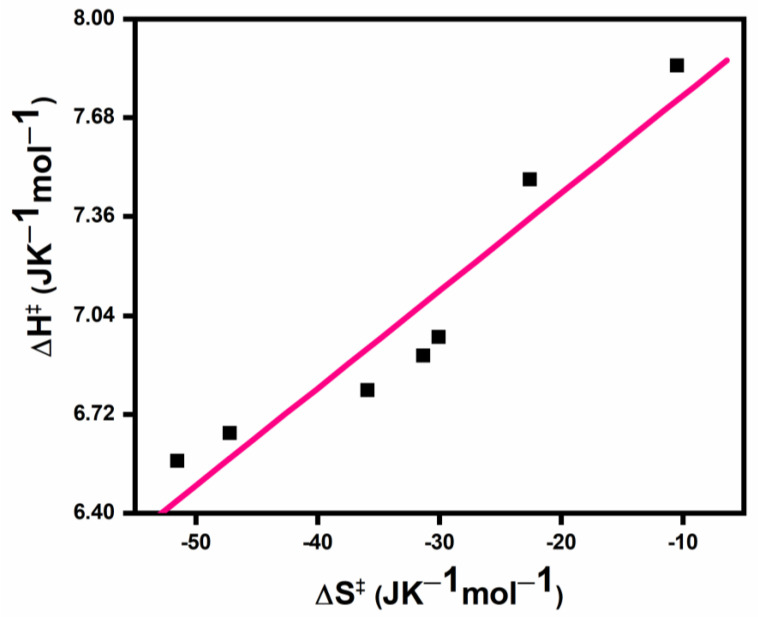
Isokinetic plot of the activation parameters for the reduction of cis-[Co(ip)_2_(C_12_H_25_NH_2_)_2_](ClO_4_)_3_ by ion(II) in [BMIM]Br medium. [Complex] = 4 × 10^−4^ mol dm^−3^; [Fe(CN)_6_]^4−^ = 0.01 mol dm^−3^; [μ] = 1.0 mol dm^−3^. (Entropy and Enthalpy of activation is symbolized ΔS^‡^ and ΔH^‡^).

In order to investigate the change in the mechanistic pathway of the OET with respect to the change in the concentration of liposome vesicles and ionic liquids at different phase transitions of oxidants, from the transition state theory [53,54], the values of entropy and enthalpy were determined by plotting ln(k/T) versus 1/T, and the plots are shown in Figure 3 and Figure 4 and SI Figures S5–S8. The ΔS^#^ and ΔH^#^ values obtained are shown in Table 3 and Table 4 and SI Tables S5–S8. As seen from these tables, the value of entropy is positive for all the reactions in both media, testifying that the formation of activated complexes is endothermic. In ionic liquids and liposome media, the entropy values are found to be negative in directions and in all the concentrations of oxidants used, indicative of a more ordered structure of the transition state. This is consistent with a model in which the oxidant and reductant bind to the liposome in the transition state. In such a case, the transition state complex attracts the surrounding water molecules, with the positive and negative charges of the ion pair leading to loss of freedom of the solvent molecules in the transition state [31]. In order to check for any change in the mechanism occurring during the electron transfer reaction with respect to the change in concentration of liposome and ionic liquids of oxidants, we have calculated the entropy and enthalpy and made isokinetic plots for the electron transfer reactions of the oxidants under study. The linearity of the plot of entropy of activation versus enthalpy of activation (Figure 5 and Figure 6 and SI Figures S9–S12) establishes the existence of a common mechanism with respect to the change in the initial concentration of oxidants.

## 4. Conclusions

In this report, the kinetics of the outer-sphere electron transfer reaction between surfactant–cobalt(III) complexes and [Fe(CN)_6_]^4−^ in DPPC and ionic liquids were studied. In DPPC liposome media, it was revealed that below the phase transition temperature, the rate decreases with increasing concentration of DPPC, which is explained by the accumulation of these surfactant–cobalt(III) complexes inside the vesicles through hydrophobic effects. Above the phase transition temperature, the rate increased with increasing concentration of DPPC, which may be due to the release of the cobalt(III) complexes from the interior to the exterior surface of the DPPC membrane. In ionic liquids media, the corresponding increase in rate constant with the increase in the concentration of ionic liquids is due to inherent amphiphilicity of (BMIM)Br which can interact with the long aliphatic chain of the surfactant–cobalt(III) complexes, therefore, the ionic liquids facilitate some aggregation of the surfactant–cobalt(III) complexes. On comparative study of the electron transfer involving ferrocyanide anion and iron(II) as reductants with the same oxidants, the rate constants involving ferrocyanide anion are higher for each of the surfactant–cobalt(III) complexes due to the good π-accepting character of ferrocyanide anion compared to iron(II). We provided the isokinetic plots for the *Cis*-[Co(ip)_2_(C_12_H_25_NH_2_)_2_]^3+^, [Co(dpq)_2_(C_12_H_25_NH_2_)_2_]^3+^ and [Co(dpqc)_2_(C_12_H_25_NH_2_)_2_]^3+^ complexes regarding ionic liquids and DPPC vesicles.

## Figures and Tables

**Table 1:** Second-order rate constants for the reduction of cobalt(III) complex ion by Fe^2+^ in DPPC medium under various temperatures; cis-[Co(ip)_2_(C_12_H_25_NH_2_)_2_](ClO_4_)_3_ = 4 × 10^−4^ mol dm^−3^, μ = 1.0 mol dm^−3^, [Fe(CN)_6_]^4−^ = 0.01 mol dm^−3^.

[DPPC] × 10^5^ (mol dm^−3^)	k × 10^2^, dm^3^ mol^−1^ s^−1^					
	298 K	303 K	308 K	323 K	328 K	333 K
2.0	15.0	15.2	15.5	16.4	16.8	17.3
3.0	14.7	15.0	15.4	17.0	17.5	18.0
4.0	14.0	14.4	15.0	17.2	17.8	18.2
5.0	13.5	13.9	14.1	17.7	18.3	18.7
6.0	12.7	13.0	13.4	18.3	18.6	19.1
7.0	11.3	11.7	12.5	18.7	19.1	19.5

**Table 2:** Second-order rate constants for the reduction of cobalt(III) complex ion by Fe^2+^ in the presence of [BMIM]Br under various temperatures; cis-[Co(ip)_2_(C_12_H_25_NH_2_)_2_](ClO_4_)_3_ = 4 × 10^−4^ mol dm^−3^, μ = 1.0 mol dm^−3^, [Fe(CN)_6_]^4−^ = 0.01 mol dm^−3^.

[(BMIM)Br] × 10^3^, mol dm^−3^	k × 10^2^, dm^−3^ mol^−1^ s^−1^					
	298 K	303 K	308 K	313 K	318 K	323 K
1.4	4.0	4.2	6.5	8.9	14.7	23.0
1.6	5.2	5.5	7.7	12.0	20.6	27.4
1.8	6.1	7.4	8.5	25.5	31.5	33.7
2.0	6.5	9.4	15.5	28.6	34.8	55.4
2.2	10.5	15.2	21.3	32.1	45.3	63.7
2.4	11.4	20.2	24.2	35.2	58.2	77.2
2.6	11.6	21.2	25.6	36.2	59.6	80.4

**Table 3:** Activation parameters for the reduction of cis-[Co(ip)_2_(C_12_H_25_NH_2_)_2_](ClO_4_)_3_, μ = 1.0 moldm^−3^ in DPPC medium.

[DPPC] × 10^5^ (mol dm^−3^)	∆H^‡^	−∆S^‡^
2.0	0.14	218.2
3.0	0.37	212.1
4.0	0.67	204.3
5.0	0.99	195.7
6.0	1.38	185.6
7.0	2.03	168.4

Entropy and Enthalpy of activation is symbolized ΔS^‡^ and ΔH^‡^.

**Table 4:** Activation parameters for the reduction of cis-[Co(ip)_2_(C_12_H_25_NH_2_)_2_](ClO_4_)_3_, μ = 1.0 moldm^−3^ in [BMIM]Br medium.

[(BMIM)Br]× 10^3^, mol dm^−3^	∆H^‡^	−∆S^‡^
1.4	6.62	50.2
1.6	6.56	49.7
1.8	6.57	42.1
2.0	6.67	35.0
2.2	6.79	34.7
2.4	7.26	24.2
2.6	7.96	7.3

Entropy and Enthalpy of activation is symbolized ΔS^‡^ and ΔH^‡^.

## Data Availability

Not applicable.

## Supplementary Materials

The following supporting information can be downloaded at: https://www.mdpi.com/article/10.3390/biomimetics7040221/s1, Figure S1: Plot of k against DPPC for Cis-[Co(dpq)_2_(C_12_H_25_NH_2_)_2_](ClO_4_)_3_ under various temperatures; cis-[Co(ip)_2_(C_12_H_25_NH_2_)_2_](ClO_4_)_3_ = 4 × 10^−4^ mol dm^−3^, μ = 1.0 mol dm^−3^, [Fe(CN)_6_ ^4−^] = 0.01 mol dm^−3^; Figure S2: Plot of k against DPPC for Cis-[Co(dpqc)_2_(C_12_H_25_NH_2_)_2_](ClO_4_)_3_ under various temperatures; cis-[Co(ip)_2_(C_12_H_25_NH_2_)_2_](ClO_4_)_3_ = 4 × 10^−4^ mol dm^−3^, μ = 1.0 mol dm^−3^, [Fe(CN)_6_ ^4−^] = 0.01 mol dm^−3^; Figure S3: Plot of k against [BMIM]Br for Cis-[Co(dpq)_2_(C_12_H_25_NH_2_)_2_](ClO_4_)_3_ at various temperatures; Cis-[Co(ip)_2_(C_12_H_25_NH_2_)_2_](ClO_4_)_3_ =4 × 10^−4^ mol dm^−3^, μ = 1.0 mol dm^−3^, [Fe(CN)_6_ ^4−^] = 0.01 mol dm^−3^; Figure S4: Plot of k against [BMIM]Br for Cis-[Co(dpqc)_2_(C_12_H_25_NH_2_)_2_](ClO_4_)_3_ at various temperatures; Cis-[Co(ip)_2_(C_12_H_25_NH_2_)_2_](ClO_4_)_3_ = 4 × 10^−4^ mol dm^−3^, μ = 1.0 mol dm^−3^, [Fe(CN)_6_ ^4−^] = 0.01 mol dm^−3^; Figure S5: Eyring plot for Cis-[Co(dpq)_2_(C_12_H_25_NH_2_)_2_](ClO_4_)_3_ in DPPC medium. [complex] = 4 × 10^−4^ mol dm^−3^; [Fe(CN)_6_ ^4−^] = 0.01 mol dm^−3^; [μ] = 1.0 mol dm^−3^; Figure S6: Eyring plot for Cis-[Co(dpqc)_2_(C_12_H_25_NH_2_)_2_](ClO_4_)_3_ in DPPC medium. [complex] = 4 × 10^−4^ mol dm^−3^; [Fe(CN)_6_ ^4−^] = 0.01 mol dm^−3^; [μ] = 1.0 mol dm^−3^; Figure S7: Eyring plot for Cis-[Co(dpq)_2_(C_12_H_25_NH_2_)_2_](ClO_4_)_3_ in [BMIM]Br medium. [complex] = 4 × 10^−4^ mol dm^−3^; [Fe(CN)_6_ ^4−^] = 0.01 mol dm^−3^; [μ] = 1.0 mol dm^−3^; Figure S8: Eyring plot for Cis-[Co(dpqc)_2_(C_12_H_25_NH_2_)_2_](ClO_4_)_3_ in [BMIM]Br medium. [complex] = 4 × 10^−4^ mol dm^−3^; [Fe(CN)_6_ ^4−^] = 0.01 mol dm^−3^; [μ] = 1.0 mol dm^−3^; Figure S9: Isokinetic plot of the activation parameters for the reduction of Cis-[Co(dpq)_2_(C_12_H_25_NH_2_)_2_](ClO_4_)_3_ by ion(II) in DPPC medium. [complex] = 4 × 10^−4^ mol dm^−3^; [Fe^2+^] = 0.01 mol dm^−3^; [μ] = 1.0 mol dm^−3^; Figure S10: Isokinetic plot of the activation parameters for the reduction of Cis-[Co(dpqc)_2_(C_12_H_25_NH_2_)_2_](ClO_4_)_3_ by ion(II) in aqueous solutions. [complex] = 4 × 10^−4^ mol dm^−3^; [Fe(CN)_6_ ^4−^] = 0.01 mol dm^−3^; [μ] = 1.0 mol dm^−3^; Figure S11: Isokinetic plot of the activation parameters for the reduction of Cis-[Co(dpq)_2_(C_12_H_25_NH_2_)_2_](ClO_4_)_3_ by ion(II) in [BMIM]Br medium. [complex] = 4 × 10^−4^ mol dm^−3^; [Fe(CN)_6_ ^4−^] = 0.01 mol dm^−3^; [μ] = 1.0 mol dm^−3^; Figure S12: Isokinetic plot of the activation parameters for the reduction of Cis-[Co(dpqc)_2_(C_12_H_25_NH_2_)_2_](ClO_4_)_3_ by ion(II) in [BMIM]Br medium. [complex] = 4 × 10^−4^ mol dm^−3^; [Fe(CN)_6_ ^4−^] = 0.01 mol dm^−3^; [μ] = 1.0 mol dm^−3^; Table S1: Second-order rate constants for the reduction of cobalt(III) complex ion by Fe^2+^ in DPPC under various temperatures. Cis-[Co(dpq)_2_(C_12_H_25_NH_2_)_2_](ClO_4_)_3_ = 4 × 10^−4^ mol dm^−3^, μ = 1.0 mol dm^−3^, [Fe(CN)_6_ ^4−^] = 0.01 mol dm^−3^; Table S2: Second-order rate constants for the reduction of cobalt(III) complex ion by Fe^2+^ in DPPC under various temperatures. Cis-[Co(dpqc)_2_(C_12_H_25_NH_2_)_2_](ClO_4_)_3_ = 4 × 10^−4^ mol dm^−3^, μ = 1.0 mol dm^−3^, [Fe(CN)_6_ ^4−^] = 0.01 mol dm^−3^;Table S3: Second-order rate constants for the reduction of cobalt(III) complex ion by Fe^2+^ in the presence of [BMIM]Br medium under various temperatures. Cis-[Co(dpq)_2_(C_12_H_25_NH_2_)_2_](ClO_4_)_3_ = 4 × 10^−4^ mol dm^−3^, μ = 1.0 mol dm^−3^, [Fe(CN)_6_ ^4−^] = 0.01 mol dm^−3^;Table S4: Second-order rate constants for the reduction of cobalt(III) complex ion by Fe^2+^ in the presence of [BMIM]Br medium under various temperatures. Cis-[Co(dpqc)_2_(C_12_H_25_NH_2_)_2_](ClO_4_)_3_ = 4 × 10^−4^ mol dm^−3^, μ = 1.0 mol dm^−3^, [Fe(CN)_6_ ^4−^] = 0.01 mol dm^−3^; Table S5: Activation parameters for the reduction of Cis-[Co(dpq)_2_(C_12_H_25_NH_2_)_2_](ClO_4_)_3_, μ = 1.0 moldm^−3^ in DPPC medium; Table S6: Activation parameters for the reduction of Cis-[Co(dpqc_2_)(C_12_H_25_NH_2_)_2_](ClO_4_)_3_, μ = 1.0 moldm^−3^ in DPPC medium; Table S7: Activation parameters for the reduction of Cis-[Co(dpq)_2_(C_12_H_25_NH_2_)_2_](ClO_4_)_3_, μ = 1.0 moldm^−3^ in DPPC medium; Table S8: Activation parameters for the reduction of Cis-[Co(dpqc)_2_(C_12_H_25_NH_2_)_2_](ClO_4_)_3_, μ = 1.0 moldm^−3^ in DPPC medium.
