# Supplementary material for: States of Aggregation and Phase Transformation Behavior of Metallosurfactant Complexes by Hexacyanoferrate(II): Thermodynamic and Kinetic Investigation of ETR in Ionic Liquids and Liposome Vesicles
Source: Biomimetics (Basel). 2022 Nov 30;7(4):221. doi: 10.3390/biomimetics7040221 (PMC9775953; doi:10.3390/biomimetics7040221)
Supplement: Supplementary file 1 [file biomimetics-07-00221-s001.zip › biomimetics-2044675-supplementary.pdf]

**Supplementary Information**

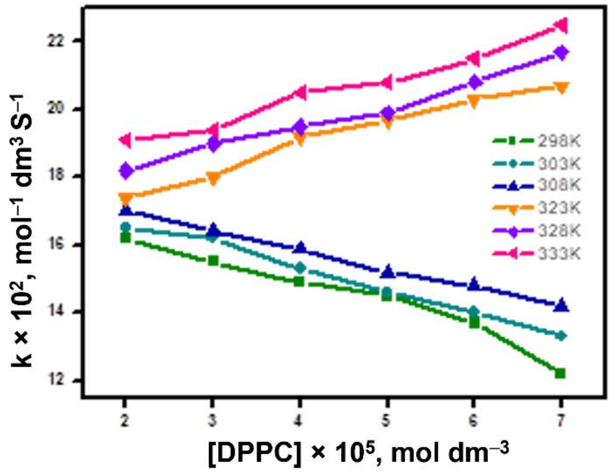


SI Figure S1 Plot of k against DPPC for Cis-[Co(dpq)$_2$($C_{12}H_{25}NH_2$)$_2$]($ClO_4$)$_3$ under various temperatures; cis-[Co(ip)$_2$($C_{12}H_{25}NH_2$)$_2$]($ClO_4$)$_3$ = 4 x $10^{-4}$ mol $dm^{-3}$, $\mu$ = 1.0 mol $dm^{-3}$, $[Fe(CN)_6]^{4-}$ = 0.01 mol $dm^{-3}$

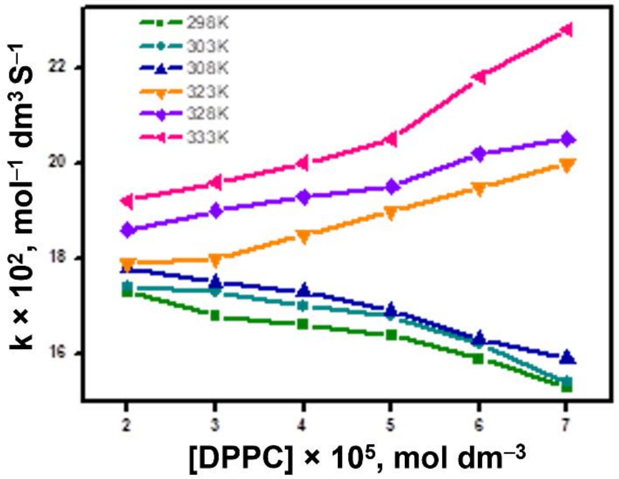


SI Figure S2 Plot of k against DPPC for Cis-[Co(dpqc)$_2$($C_{12}H_{25}NH_2$)$_2$]($ClO_4$)$_3$ under various temperatures; cis-[Co(ip)$_2$($C_{12}H_{25}NH_2$)$_2$]($ClO_4$)$_3$ = 4 x $10^{-4}$ mol $dm^{-3}$, $\mu$ = 1.0 mol $dm^{-3}$, $[Fe(CN)_6]^{4-}$ = 0.01 mol $dm^{-3}$

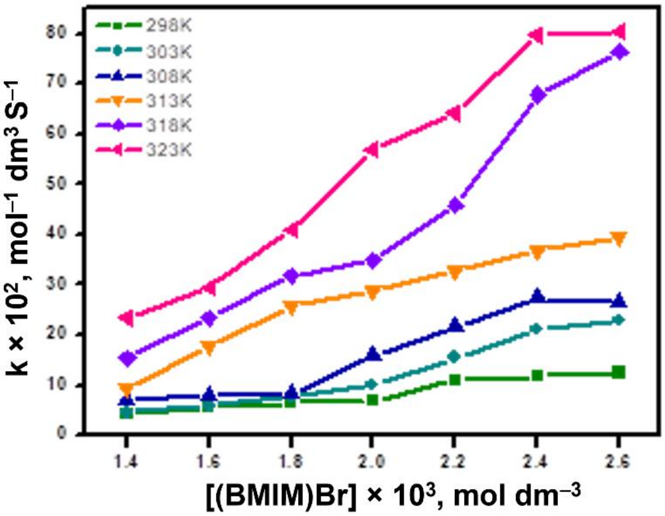


SI Figure S3 Plot of k against [BMIM]Br for Cis-$[Co(dpq)_2(C_{12}H_{25}NH_2)_2](ClO_4)_3$ at various temperatures; Cis-$[Co(ip)_2(C_{12}H_{25}NH_2)_2](ClO_4)_3$ =4 x $10^{-4}$ mol $dm^{-3}$, $\mu$ = 1.0 mol $dm^{-3}$, $[Fe(CN)_6]^{4-}$ = 0.01 mol $dm^{-3}$

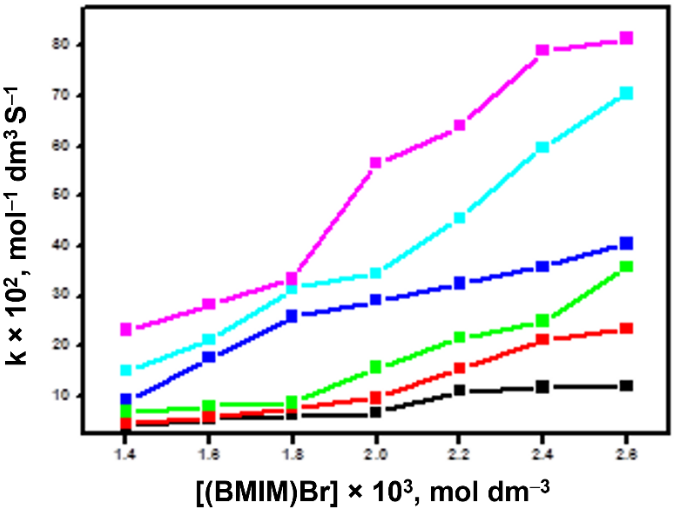


SI Figure S4 Plot of k against [BMIM]Br for Cis-$[Co(dpqc)_2(C_{12}H_{25}NH_2)_2](ClO_4)_3$ at various temperatures; Cis-$[Co(ip)_2(C_{12}H_{25}NH_2)_2](ClO_4)_3$ = 4 x $10^{-4}$ mol $dm^{-3}$, $\mu$ = 1.0 mol $dm^{-3}$, $[Fe(CN)_6]^{4-}$ = 0.01 mol $dm^{-3}$

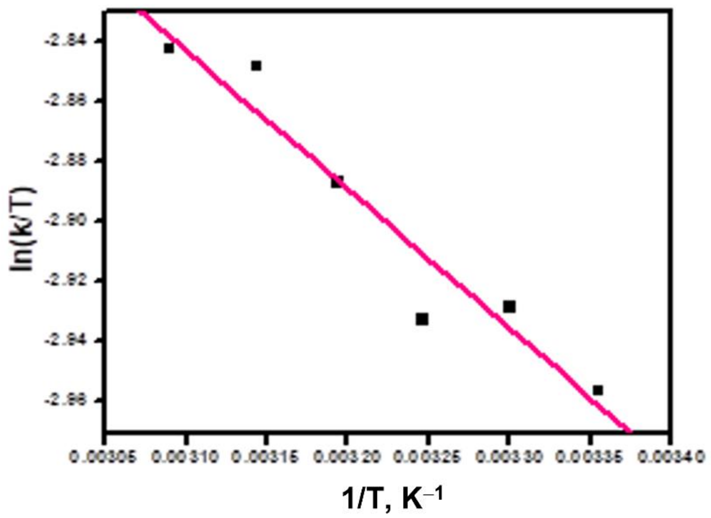


SI Figure S5 Eyring plot for Cis-$[Co(dpq)_2(C_{12}H_{25}NH_2)_2](ClO_4)_3$ in DPPC medium. [complex] = 4 x $10^{-4}$ mol $dm^{-3}$; $[Fe(CN)_6]^{4-}$ = 0.01 mol $dm^{-3}$; [μ] = 1.0 mol $dm^{-3}$.

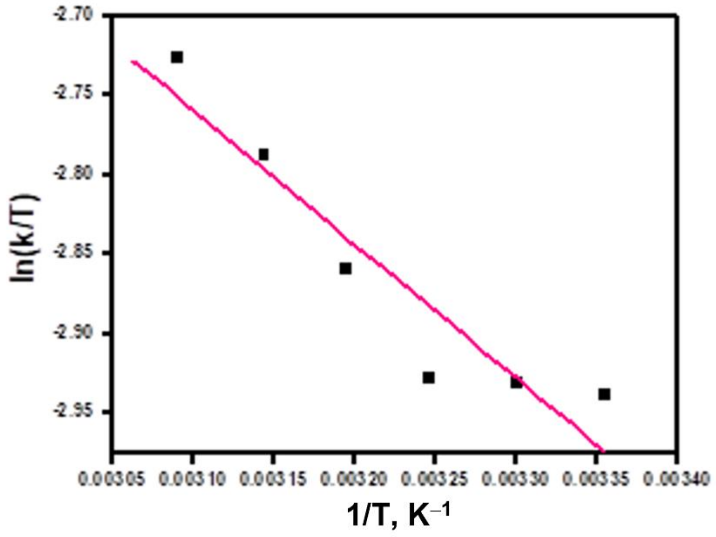


SI Figure S6 Eyring plot for Cis-$[Co(dpqc)_2(C_{12}H_{25}NH_2)_2](ClO_4)_3$ in DPPC medium. [complex] = 4 x $10^{-4}$ mol $dm^{-3}$; $[Fe(CN)_6]^{4-}$ = 0.01 mol $dm^{-3}$; [μ] = 1.0 mol $dm^{-3}$.

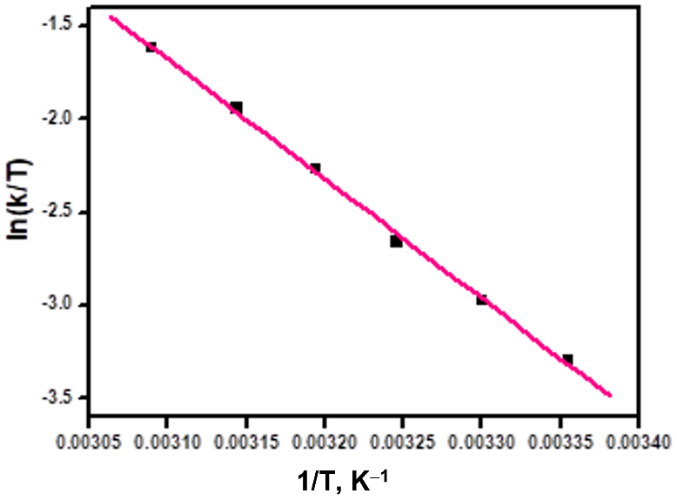


SI Figure S7 Eyring plot for Cis-[Co(dpq)$_2$(C$_{12}$H$_{25}$NH$_2$)$_2$](ClO$_4$)$_3$ in [BMIM]Br medium. [complex] = 4 x 10$^{-4}$ mol dm$^{-3}$; [Fe(CN)$_6$]$^{4-}$ = 0.01 mol dm$^{-3}$; [μ] = 1.0 mol dm$^{-3}$.

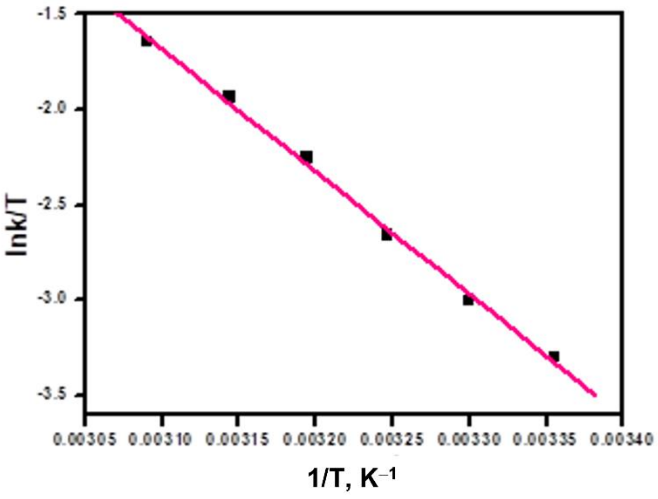


SI Figure S8 Eyring plot for Cis-[Co(dpqc)$_2$(C$_{12}$H$_{25}$NH$_2$)$_2$](ClO$_4$)$_3$ in [BMIM]Br medium. [complex] = 4 x 10$^{-4}$ mol dm$^{-3}$; [Fe(CN)$_6$]$^{4-}$ = 0.01 mol dm$^{-3}$; [μ] = 1.0 mol dm$^{-3}$.

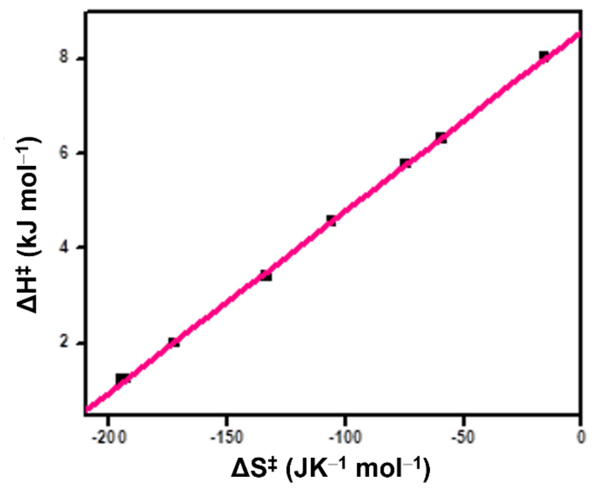


SI Figure S9 Isokinetic plot of the activation parameters for the reduction of Cis-[Co(dpq)$_2$($C_{12}H_{25}NH_2$)$_2$]($ClO_4$)$_3$ by ion(II) in DPPC medium. [complex] = 4 x $10^{-4}$ mol $dm^{-3}$; [$Fe^{2+}$] = 0.01 mol $dm^{-3}$; [μ] = 1.0 mol $dm^{-3}$. Note: Entropy and Enthalpy of activation is symbolized $\Delta S^{\ddagger}$ and $\Delta H^{\ddagger}$.

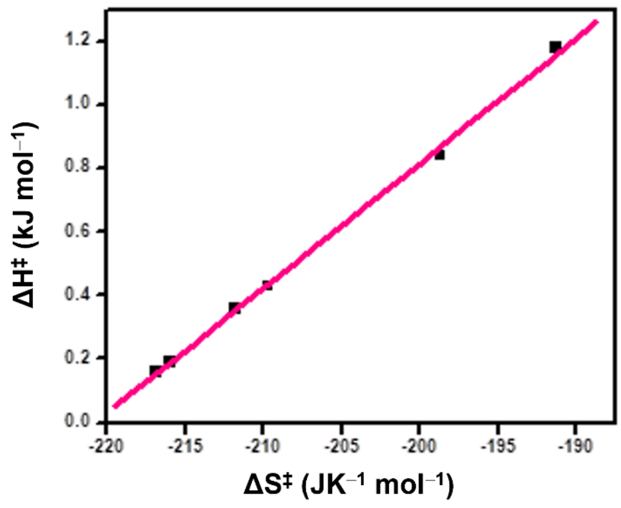


SI Figure S10 Isokinetic plot of the activation parameters for the reduction of Cis-[Co(dpqc)$_2$($C_{12}H_{25}NH_2$)$_2$]($ClO_4$)$_3$ by ion(II) in aqueous solutions. [complex] = 4 x $10^{-4}$ mol $dm^{-3}$; [$Fe(CN)_6$]$^{4-}$ = 0.01 mol $dm^{-3}$; [μ] = 1.0 mol $dm^{-3}$. Note: Entropy and Enthalpy of activation is symbolized $\Delta S^{\ddagger}$ and $\Delta H^{\ddagger}$.

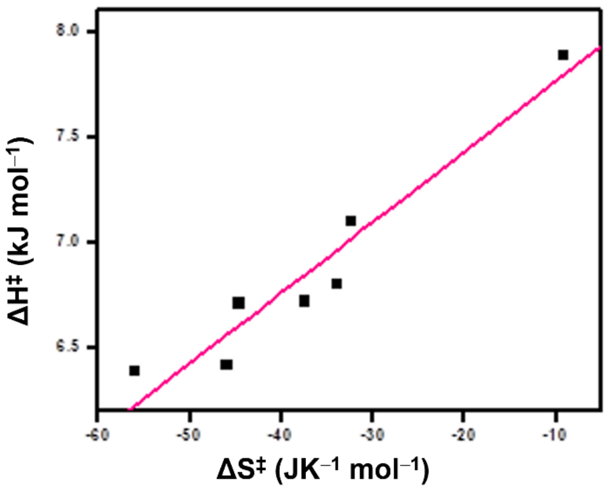


SI Figure S11 Isokinetic plot of the activation parameters for the reduction of Cis-[Co(dpq)$_2$(C$_{12}$H$_{25}$NH$_2$)$_2$](ClO$_4$)$_3$ by ion(II) in [BMIM]Br medium. [complex] = 4 x 10$^{-4}$ mol dm$^{-3}$; [Fe(CN)$_6$]$^{4-}$ = 0.01 mol dm$^{-3}$; [μ] = 1.0 mol dm$^{-3}$. Note: Entropy and Enthalpy of activation is symbolized ΔS$^‡$ and ΔH$^‡$.

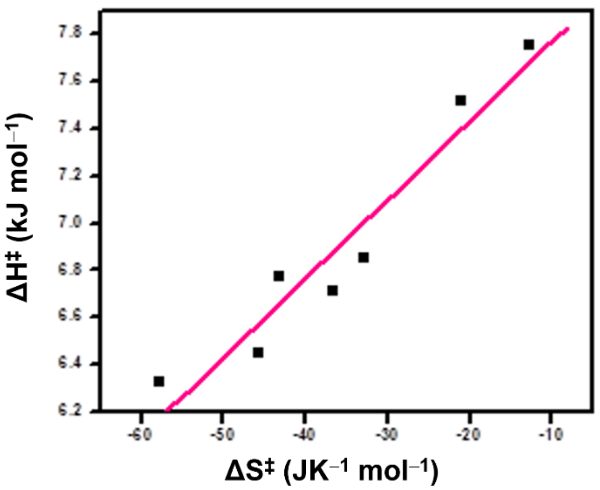


SI Figure S12 Isokinetic plot of the activation parameters for the reduction of Cis-[Co(dpqc)$_2$(C$_{12}$H$_{25}$NH$_2$)$_2$](ClO$_4$)$_3$ by ion(II) in [BMIM]Br medium. [complex] = 4 x 10$^{-4}$ mol dm$^{-3}$; [Fe(CN)$_6$]$^{4-}$ = 0.01 mol dm$^{-3}$; [μ] = 1.0 mol dm$^{-3}$. **Note:** Entropy and Enthalpy of activation is symbolized ΔS$^‡$ and ΔH$^‡$.

## Tables

SI Table S1. Second-order rate constants for the reduction of cobalt(III) complex ion by $Fe^{2+}$ in DPPC under various temperatures. Cis-$[Co(dpq)_2(C_{12}H_{25}NH_2)_2](ClO_4)_3$ = 4 x $10^{-4}$ mol $dm^{-3}$, μ = 1.0 mol $dm^{-3}$, $[Fe(CN)_6]^{4-}$ = 0.01 mol $dm^{-3}$

| **[DPPC] × $10^5$ (mol $dm^{-3}$)** | **k ×$10^2$, $dm^3$ $mol^{-1}$ $s^{-1}$** | | | | | |
|---|---|---|---|---|---|---|
| | 298K | 303K | 308K | 323K | 328K | 333K |
| 2.0 | 16.2 | 16.5 | 17.0 | 17.4 | 18.2 | 19.1 |
| 3.0 | 15.5 | 16.2 | 16.4 | 18.0 | 19.0 | 19.4 |
| 4.0 | 14.9 | 15.3 | 15.9 | 19.2 | 19.5 | 20.5 |
| 5.0 | 14.5 | 14.6 | 15.2 | 19.7 | 19.9 | 20.8 |
| 6.0 | 13.7 | 14.0 | 14.8 | 20.3 | 20.8 | 21.5 |
| 7.0 | 12.2 | 13.3 | 14.2 | 20.7 | 21.7 | 22.5 |

SI Table S2. Second-order rate constants for the reduction of cobalt(III) complex ion by $Fe^{2+}$ in DPPC under various temperatures. Cis-$[Co(dpqc)_2(C_{12}H_{25}NH_2)_2](ClO_4)_3$ = 4 x $10^{-4}$ mol $dm^{-3}$, μ = 1.0 mol $dm^{-3}$, $[Fe(CN)_6]^{4-}$ = 0.01 mol $dm^{-3}$

| **[DPPC] × $10^5$ (mol $dm^{-3}$)** | **k ×$10^2$, $dm^3$ $mol^{-1}$ $s^{-1}$** | | | | | |
|---|---|---|---|---|---|---|
| | 298K | 303K | 308K | 323K | 328K | 333K |
| 2.0 | 17.3 | 17.4 | 17.8 | 17.9 | 18.6 | 19.2 |
| 3.0 | 16.8 | 17.3 | 17.5 | 18.0 | 19.0 | 19.6 |
| 4.0 | 16.6 | 17.0 | 17.3 | 18.5 | 19.3 | 20.0 |
| 5.0 | 16.4 | 16.8 | 16.9 | 19.0 | 19.5 | 20.5 |
| 6.0 | 15.9 | 16.2 | 16.3 | 19.5 | 20.2 | 21.8 |
| 7.0 | 15.3 | 15.4 | 15.9 | 20.0 | 20.5 | 22.8 |

SI Table S3. Second-order rate constants for the reduction of cobalt(III) complex ion by $Fe^{2+}$ in the presence of [BMIM]Br medium under various temperatures. Cis-$[Co(dpq)_2(C_{12}H_{25}NH_2)_2](ClO_4)_3$ = 4 x $10^{-4}$ mol $dm^{-3}$, μ = 1.0 mol $dm^{-3}$, $[Fe(CN)_6]^{4-}$ = 0.01 mol $dm^{-3}$

| **[(BMIM)Br] × $10^3$, mol $dm^{-3}$** | **k ×$10^2$, $dm^3$ $mol^{-1}$ $s^{-1}$** | | | | | |
|---|---|---|---|---|---|---|
| | 298K | 303K | 308K | 313K | 318K | 323K |
| 1.4 | 4.0 | 4.2 | 6.5 | 8.9 | 14.7 | 23.0 |
| 1.6 | 5.2 | 5.5 | 7.7 | 12.0 | 20.6 | 27.4 |
| 1.8 | 6.1 | 7.4 | 8.5 | 25.5 | 31.5 | 33.7 |
| 2.0 | 6.5 | 9.4 | 15.5 | 28.6 | 34.8 | 55.4 |
| 2.2 | 10.5 | 15.2 | 21.3 | 32.1 | 45.3 | 63.7 |
| 2.4 | 11.4 | 20.2 | 24.2 | 35.2 | 58.2 | 77.2 |
| 2.6 | 11.6 | 21.2 | 25.6 | 36.2 | 59.6 | 80.4 |

SI Table S4. Second-order rate constants for the reduction of cobalt(III) complex ion by $Fe^{2+}$ in the presence of [BMIM]Br medium under various temperatures. Cis-$[Co(dpqc)_2(C_{12}H_{25}NH_2)_2](ClO_4)_3$ = 4 x $10^{-4}$ mol $dm^{-3}$, μ = 1.0 mol $dm^{-3}$, $[Fe(CN)_6]^{4-}$ = 0.01 mol $dm^{-3}$

| **[(BMIM)Br] ×$10^3$, mol $dm^{-3}$** | **k ×$10^2$, $dm^{-3}$ $mol^{-1}$ $s^{-1}$** | | | | | |
|---|---|---|---|---|---|---|
| | 298K | 303K | 308K | 313K | 318K | 323K |
| 1.4 | 4.3 | 4.6 | 7.0 | 9.5 | 15.4 | 23.5 |
| 1.6 | 5.6 | 5.9 | 8.1 | 17.8 | 23.5 | 29.5 |
| 1.8 | 6.5 | 7.6 | 8.3 | 25.9 | 31.8 | 41.0 |
| 2.0 | 6.8 | 10.0 | 15.9 | 29 | 35.0 | 57.0 |
| 2.2 | 11.0 | 15.5 | 21.6 | 32.9 | 45.9 | 64.2 |
| 2.4 | 11.8 | 21.1 | 27.4 | 36.9 | 67.8 | 80.8 |
| 2.6 | 12.5 | 22.9 | 26.5 | 39.5 | 76.4 | 82.4 |

SI Table S5. Activation parameters for the reduction of Cis-$[Co(dpq)_2(C_{12}H_{25}NH_2)_2](ClO_4)_3$ , $\mu$ = 1.0 moldm$^{-3}$ in DPPC medium. **Note:** Entropy and Enthalpy of activation is symbolized $\Delta S^‡$ and $\Delta H^‡$.

| **[DPPC]×10$^5$ (mol dm$^{-3}$)** | **$\Delta H^‡$** | **-$\Delta S^‡$** |
|---|---|---|
| 2.0 | 1.26 | 193.3 |
| 3.0 | 2.01 | 172.5 |
| 4.0 | 3.43 | 133.4 |
| 5.0 | 4.57 | 106.0 |
| 6.0 | 5.79 | 74.20 |
| 7.0 | 6.33 | 59.40 |
| 8.0 | 8.02 | 15.80 |

SI Table S6. Activation parameters for the reduction of Cis-$[Co(dpqc_2(C_{12}H_{25}NH_2)_2](ClO_4)_3$ , $\mu$ = 1.0 moldm$^{-3}$ in DPPC medium. **Note:** Entropy and Enthalpy of activation is symbolized $\Delta S^‡$ and $\Delta H^‡$.

| **[DPPC]×10$^5$ (mol dm$^{-3}$)** | **$\Delta H^‡$** | **-$\Delta S^‡$** |
|---|---|---|
| 2.0 | 0.16 | 216.9 |
| 3.0 | 0.19 | 216.0 |
| 4.0 | 0.36 | 211.8 |
| 5.0 | 0.43 | 209.7 |
| 6.0 | 0.84 | 198.7 |
| 7.0 | 1.18 | 191.3 |

SI Table S7. Activation parameters for the reduction of Cis-[Co(dpq)$_2$($C_{12}H_{25}NH_2$)$_2$]($ClO_4$)$_3$ , $\mu$ = 1.0 moldm$^{-3}$ in [BMIM]Br medium. **Note:** Entropy and Enthalpy of activation is symbolized $\Delta S^{\ddagger}$ and $\Delta H^{\ddagger}$.

| **[(BMIM)Br]× $10^3$, mol dm$^{-3}$** | **$\Delta H^{\ddagger}$** | **-$\Delta S^{\ddagger}$** |
|---|---|---|
| 1.4 | 6.39 | 55.9 |
| 1.6 | 6.42 | 45.9 |
| 1.8 | 6.71 | 44.6 |
| 2.0 | 6.72 | 37.4 |
| 2.2 | 6.80 | 33.9 |
| 2.4 | 7.10 | 32.4 |
| 2.6 | 7.89 | -9.2 |

SI Table S8. Activation parameters for the reduction of Cis-[Co(dpqc)$_2$($C_{12}H_{25}NH_2$)$_2$]($ClO_4$)$_3$ , $\mu$ = 1.0 moldm$^{-3}$ in [BMIM]Br medium. **Note:** Entropy and Enthalpy of activation is symbolized $\Delta S^{\ddagger}$ and $\Delta H^{\ddagger}$.

| **[(BMIM)Br]× $10^3$, mol dm$^{-3}$** | **$\Delta H^{\ddagger}$** | **-$\Delta S^{\ddagger}$** |
|---|---|---|
| 1.4 | 6.33 | 57.7 |
| 1.6 | 6.45 | 45.7 |
| 1.8 | 6.77 | 43.2 |
| 2.0 | 6.71 | 36.5 |
| 2.2 | 6.85 | 32.8 |
| 2.4 | 7.52 | 20.9 |
| 2.6 | 7.75 | 12.6 |
